# Estimation of Asthma Symptom Onset Using Internet Search Queries: Lag-Time Series Analysis

**DOI:** 10.2196/18593

**Published:** 2021-05-10

**Authors:** Yulin Hswen, Amanda Zhang, Bruno Ventelou

**Affiliations:** 1 Department of Epidemiology and Biostatistics Bakar Computational Health Sciences Institute University of California San Francisco San Francisco, CA United States; 2 Aix Marseille University, CNRS, AMSE Marseille France; 3 Mathematics Department Harvard University Cambridge, MA United States

**Keywords:** digital epidemiology, Google queries, asthma, symptoms, health information seeking

## Abstract

**Background:**

Asthma affects over 330 million people worldwide. Timing of an asthma event is extremely important and lack of identification of asthma increases the risk of death. A major challenge for health systems is the length of time between symptom onset and care seeking, which could result in delayed treatment initiation and worsening of symptoms.

**Objective:**

This study evaluates the utility of the internet search query data for the identification of the onset of asthma symptoms.

**Methods:**

Pearson correlation coefficients between the time series of hospital admissions and Google searches were computed at lag times from 4 weeks before hospital admission to 4 weeks after hospital admission. An autoregressive integrated moving average (ARIMAX) model with an autoregressive process at lags of 1 and 2 and Google searches at weeks –1 and –2 as exogenous variables were conducted to validate our correlation results.

**Results:**

Google search volume for asthma had the highest correlation at 2 weeks before hospital admission. The ARIMAX model using an autoregressive process showed that the relative searches from Google about asthma were significant at lags 1 (*P*<.001) and 2 (*P*=.04).

**Conclusions:**

Our findings demonstrate that internet search queries may provide a real-time signal for asthma events and may be useful to measure the timing of symptom onset.

## Introduction

Asthma is a significant contributor to disease burden globally [1]. It kills around 1000 people every day and affects over 330 million people worldwide, a number that continues to rise [2]. A major challenge for health systems is the length of time between symptom onset and care seeking, which could result in delayed treatment initiation and worsening of symptoms [3,4]. Consequently, the World Health Organization has prioritized reducing asthma burden, as over 330 million people have asthma, and it is also the most common chronic disease of childhood. Avoidable asthma deaths still occur due to lack of early identification and inappropriate management [5]. Date of hospitalization has been used in the majority of time-series studies because it is the only available administrative data on the timing of the asthma event. However, evidence has emerged that assessment exposure based on hospitalization data may generate measurement bias and lead to misclassification of time of event onset [4]. The true onset of the symptomatic event may have occurred days prior to hospital admission, leading to underestimation of the strength of association between environmental exposures such as ambient air pollution and acute clinical asthma events [3,6,7]. This is of particular concern for asthma because of its acute event onset and because it is sensitive to short-term ambient air pollution fluctuations.

**Figure 1:**
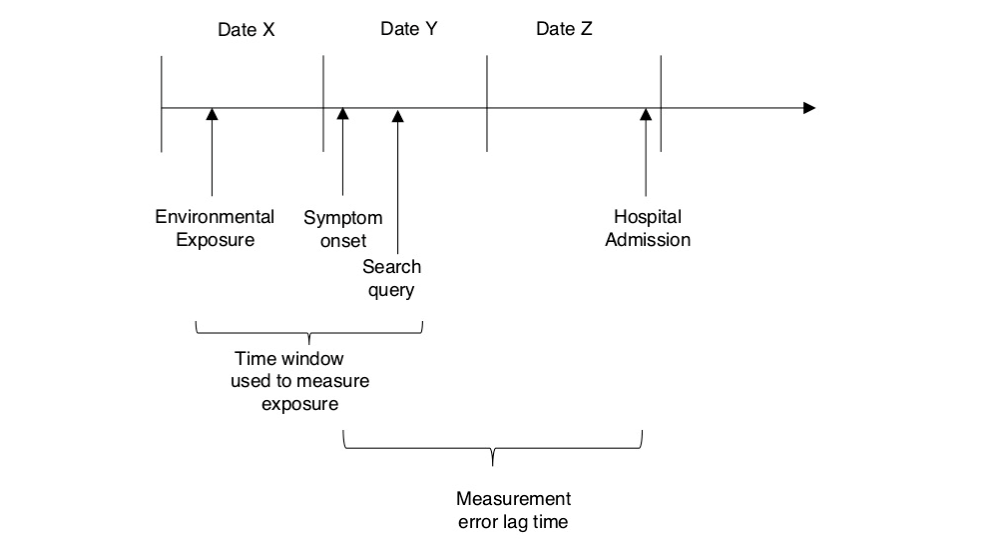
Potential difference in lag time between true time of onset and hospital admission. Diagrammatic representation of exposure measurement error. Date of hospital admission is not necessarily the date of symptom onset and may lead to misclassification, as exposure measurement does not fall within the period of hospital admission. Internet search queries may identify onset of symptoms in real time and earlier than administrative data and reduce measurement error.

Web searching has become integral for finding health-related information. Existing evidence shows that individuals use search engines to understand their health symptoms, especially at earlier stages of their illness, before making a medical visit or use the web to decide whether to admit themselves to a health care center [8,9]. Some individuals even use information gathered from the internet to make decisions on how to treat their illness as opposed to visiting a provider [10]. Based on these information-searching behaviors, researchers have utilized internet search queries for early identification of disease onset, which has shown to be effective for the detection of infectious disease epidemics including influenza and Ebola [11-13]. However, research has yet to evaluate the potential utility of search queries to identify the onset of asthma symptoms and minimize measurement bias (Figure 1). This study examines whether internet search queries could reveal the lag time between onset of asthma symptoms and hospital admissions due to asthma events.

## Methods

### Overview

To investigate the ability of internet search queries to detect the time of onset, we analyzed the lag time between internet search activity and asthma-related hospital visits in the Provence-Alpes-Côte d’Azur (PACA) region in France. France’s health care system gives the rare opportunity to make a complete account of all admissions in a given territory (in both public and private hospitals) [14]. PACA, France, was chosen to be the focus for this study because of its high national emissions of air pollution [15,16]. The number of asthma-related hospitalizations (International Classification of Diseases [ICD]-10 codes J45 and J46) was collected from diagnosis-related group (DRG)–based Program for Medicalization of Information Systems (PMSI) from all the hospitals in the PACA region and aggregated at the weekly level [17].

### Google Relative Search Volumes

Time series of weekly Google relative search volumes (RSVs) for the term topic “asthme” (asthma) restricted to the PACA region were collected from January 1, 2017, to December 31, 2017, from Google Trends [18]. Google computes RSVs by dividing the total search volume for a query in a given geographical location by the total number of queries in that region at a given point in time [19]. Therefore, these data are normalized by the population density and search volume in a given geographical area and account for temporal fluctuations. This means that when we look at the search interest for the topic of asthma, it will be proportional to all searches on all topics on Google at that time and location. This function allowed us to measure the overall interest in the topic related to asthma in this study.

Because we were specifically interested in asthma hospital admissions in the PACA region in France, other related terms, such as “difficulty breathing,” were not used, as they are not specific to asthma and overlap with other respiratory conditions. Search queries related to the topic term “saignement” (bleeding) were collected as a control, as bleeding has no direct medical connection to asthma. Pearson correlation coefficients between the time series of hospital admissions and Google searches were computed at lag times from 4 weeks before hospital admission to 4 weeks after hospital admission. We further tested the Pearson correlation results with an autoregressive analysis using explanatory variables: autoregressive integrated moving average (ARIMAX) with an autoregressive process at lags of 1 and 2 and Google searches at weeks –1 and –2 weeks as exogenous variables (Table 1). This allowed us to assess how Google searches were associated with hospital admissions while accounting for autocorrelation of the hospital admissions time series. The data that support the findings of this study were obtained from Google Trends that are available from [18] and from the DRG-based PMSI under a license for this study and are not publicly available; however, these can be obtained from the authors upon reasonable request and with permission of the DRG-based PMSI. All analyses were conducted using the statsmodels package in Python.

**Table 1:** ARIMAX^a^ regression.

Variable	Coefficient	Standard error	*P* value	95% CI
AR^b^-1 (hospital admissions 1 week ago)	0.83	1.15	<.001	0.41 to 1.26
AR-2 (hospital admissions 2 weeks ago)	–0.31	1.05	.04	–0.59 to –0.02
“asthme” Google searches 1 week ago	3.67	0.22	.001	1.42 to 5.92
“asthme” Google searches 2 weeks ago	3.59	0.15	.001	1.52 to 5.65
Variance on error term	461.84	81.83	<.001	301.46 to 622.22

^a^ARIMAX: autoregressive integrated moving average.

^b^AR: autoregression.

### Ethics and Consent

Public use data sets used in this study are in aggregate format and not individually identifiable such that their analysis is deemed nonhuman subject research.

## Results

**Figure 2:**
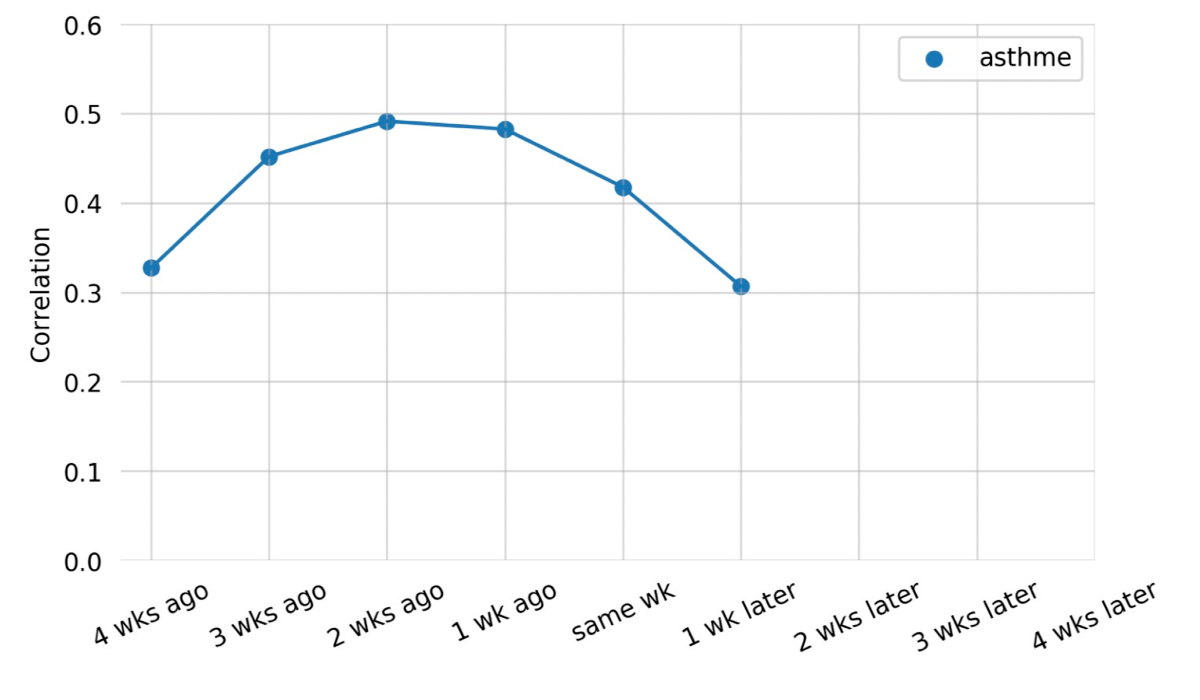
Lag correlation between searches and admissions for “asthme,” 2017.

Google RSVs for asthma had the highest correlation at 2 weeks before admission with a correlation of 0.491 (*P*<.001; Figure 2, Table 2). Searches for “saignement” (bleeding) did not exhibit significant positive correlations with asthma-related hospital admissions at any lag time (Table 2). Our results of the Pearson correlation were further validated with our ARIMAX model, whereby the relative Google searches about asthma were significant at 1 (*P*<.001) and 2 (*P*=.004) weeks’ lags before hospital admissions, which were consistent with our correlation results.

**Table 2:** Pearson correlations between Google search term and hospital admissions for asthma.

Lag time	“Asthme” (asthma) searches × asthma admissions			“Saignement” (bleeding) searches × asthma admissions	
	Correlation	*P* value	Correlation		*P* value
4 weeks before	0.327	.02	0.139		.34
3 weeks before	0.452	.001	0.123		.39
2 weeks before	0.491	<.001	0.046		.74
1 week before	0.483	<.001	–0.046		.74
Same week	0.418	.002	–0.034		.80
1 week after	0.307	.02	–0.082		.56
2 weeks after	0.013	.93	–0.220		.12
3 weeks after	–0.261	.07	–0.248		.08
4 weeks after	-0.426	.003	–0.268		.06

## Discussion

### Principal Findings

Results from our study suggest that internet search queries detect asthma symptom onset earlier than hospital admissions. Delay between time of symptom onset and time of hospital presentation for acute clinical events has been shown to result in considerable underestimation of the effects of ambient air pollution [4]. Our results show the greatest correlation at a lag of 2 weeks between Google RSVs for asthma and asthma hospital visits. In comparison to a recent study that tested the lag time between Google RSVs for terms related to COVID-19 and COVID-19 cases in Taiwan, significant time-lag correlations ranged from 0.33 to 0.72 [20]. Results from our time-series correlation were in between the range of these correlations at 0.49 (*P*<.001) at 2 weeks. This effect size of 0.49 indicates a moderate relationship as correlations over 0.3 are considered to indicate an underlying relationship between 2 variables of interest [21,22]. The 1- and 2-week lag we found between asthma searches and asthma hospitalizations is consistent with previous studies that have on average a 2-week time lag between internet-based and traditional surveillance systems for disease surveillance [23]. In a study that looked at internet searches on dengue fever and local dengue occurrences, a lag time of 1 week was reported [24]. In a more recent study, the relationship between chest pain search volume on Google and new COVID-19 cases saw a lag time of 18 days (2-3 weeks) [25]. This consistent time lag of around 2 weeks may indicate the amount of time that elapsed between users developing symptoms and seeking in-person medical care.

Our results highlight that online internet search queries about symptoms may offer a novel approach to (1) identify the timing and the magnitude of future admissions, to prepare and manage resources efficiently at the hospitals (as suggested for opioids [10]) and (2) correct for measurement bias and misclassification of time of asthma onset. This finding is important, as short-term fluctuations in ambient air pollution can have significant effects on acute symptoms; therefore, hour-by-hour estimates are important to understand the impact of these environmental exposures on symptomatic changes in order to identify the onset of larger more severe health events. Epidemiological studies measuring exposure and response should investigate the lag time between search queries and hospitalization to uncover insights about the actual timing of onset. Recent evidence has also indicated that the COVID-19 pandemic has led patients to use internet search on Google to seek out medial information and treatment in replacement of professional medical attention [25]. For instance, compared with previous years, there have been significant reductions in hospital presentations for acute myocardial infarctions and concurrent increases in out-of-hospital cardiac arrests during the COVID-19 pandemic and a marked spike in search volume for chest pain [26,27]. Therefore, internet search queries related to respiratory symptoms may offer insight into the true incidence of respiratory illnesses during COVID-19, as fear of contracting COVID-19 may prevent patients from seeking hospital care. Future studies should use internet search queries to estimate the incidence of disease, as hospital admissions may not be able to provide accurate measurements in the time of COVID-19.

### Limitations

We recognize that the population on the search engine Google may not be entirely representative of the French population. However, statistics show that Google holds the largest market share of all search engines in France (92% as of September 2020) [28]. The population of internet users in France is 82.0% and skewed toward younger age and higher education level [29]. In this study, we validated that search strategies were effective at identifying the onset of future emergency hospital use. Despite the limitation that searches on Google might not be generalizable to the entire French population, our results still suggest that this methodology may be applicable to other chronic diseases as well. However, we acknowledge that this method may not be applicable to all types of symptoms or hospital uses, especially when the disease to be treated is very specific to a subpopulation.

We also recognize that recent developments in time series such as time-series forecasting were not used for our analysis. However, in this study we sought to model trends in searches as they related to external factors such as emergency visits, whereas time-series forecasting seeks to forecast future values of that series such as using historical hospital visits to predict future hospital visits. We also chose to use the most standard and frequently used time-series model for consistency in the research area related to environmental respiratory disease in order to identify the time lag between symptom onset and hospital admission [30-33].

### Future Directions

Based on our study findings, we believe that earlier identification of potential cases of asthma exacerbation through internet searches could help improve the efficiency of resource allocation within hospitals such as staff, beds, and respiratory assistance. Future studies should test the ability of Google searches in the hospital setting to predict cases and reduce the burden on hospitals. In addition, since the COVID-19 pandemic, it has been postulated that many patients are not seeking care for their arising symptoms because of fears of COVID-19 transmission [27]. Therefore, the use of internet search could help identify real-time and accurate onset of asthma during the time of the COVID-19 pandemic. This information can be used to provide timely and correct patient education including informing the public about the appropriate course of action. Public health efforts should consider the utility of internet searches for respiratory conditions such as asthma to measure care-seeking behaviors and prevent severe long-term consequences.

### Conclusions

Asthma is one of the most significant noncommunicable diseases globally [34]. Improving surveillance is crucial for the control of asthma and the prevention of avoidable deaths due to this disease. The use of online digital surveillance offers the ability to capture the onset of asthma more accurately and rapidly and has the potential to reduce the burden and deaths caused by asthma.

## Abbreviations

ARIMAX: autoregressive integrated moving average
DRG: diagnosis-related group
ICD: International Classification of Diseases
PACA: Provence-Alpes-Côte d’Azur
PMSI: Program for Medicalization of Information Systems
RSV: relative search volumes
